# Bis[μ_2_-bis­(diphenyl­phosphino)methane]bis­(μ_2_-ethane-1,2-dithiol­ato)-μ_4_-sulfido-μ_2_-sulfido-disulfidodimolybdenum(V)disilver(I) dimethyl­formamide tris­olvate

**DOI:** 10.1107/S1600536809048648

**Published:** 2009-11-21

**Authors:** Xing-Cong Wang, Xiu-Li You

**Affiliations:** a Jiangxi Key Laboratory of Organic Chemistry, Jiangxi Science and Technology Normal University, Nanchang 330013, People’s Republic of China

## Abstract

Treatment of [Et_4_N]_2_[(edt)_2_Mo_2_S_2_(μ-S)_2_] (edt = ethane­dithio­l­ate) with two equivalents of Ag(CH_3_CN)_4_ClO_4_ in the presence of bis­(diphenyl­phosphino)methane (dppm) ligand gives rise to the title tetra­nuclear cluster, [Ag_2_Mo_2_(C_2_H_4_S_2_)_2_S_4_(C_25_H_22_P_2_)_2_]·3C_3_H_7_NO. The complex mol­ecule and one of the dimethyl­formamide (DMF) solvent mol­ecules occupy special positions on a mirror plane. The mol­ecular structure of the complex may be visualized as being built of [Mo_2_S_2_(μ-S)_2_(edt)_2_]^2−^ dianions and [Ag_2_(dppm)_2_]^2+^ dications connected by two Ag—μ-S_edt_ and two Ag—μ_4_-S bonds.

## Related literature

For general background to the chemistry of sulfido-bridged dinuclear clusters consisting of a [*M*_2_S_4_] core (*M* = Mo, W) and various transition metals, see: Kuwata & Hidai (2001 ▶); Curtis *et al.* (1997 ▶); Halbert *et al.* (1985 ▶); Kawaguchi *et al.* (1997 ▶); Brunner *et al.* (1985 ▶). For the synthesis and structure of the starting material, see: Pan *et al.* (1984 ▶). For related structures, see: Zhu *et al.* (1990 ▶); Lin *et al.* (1997 ▶); Wei *et al.* (2008 ▶).
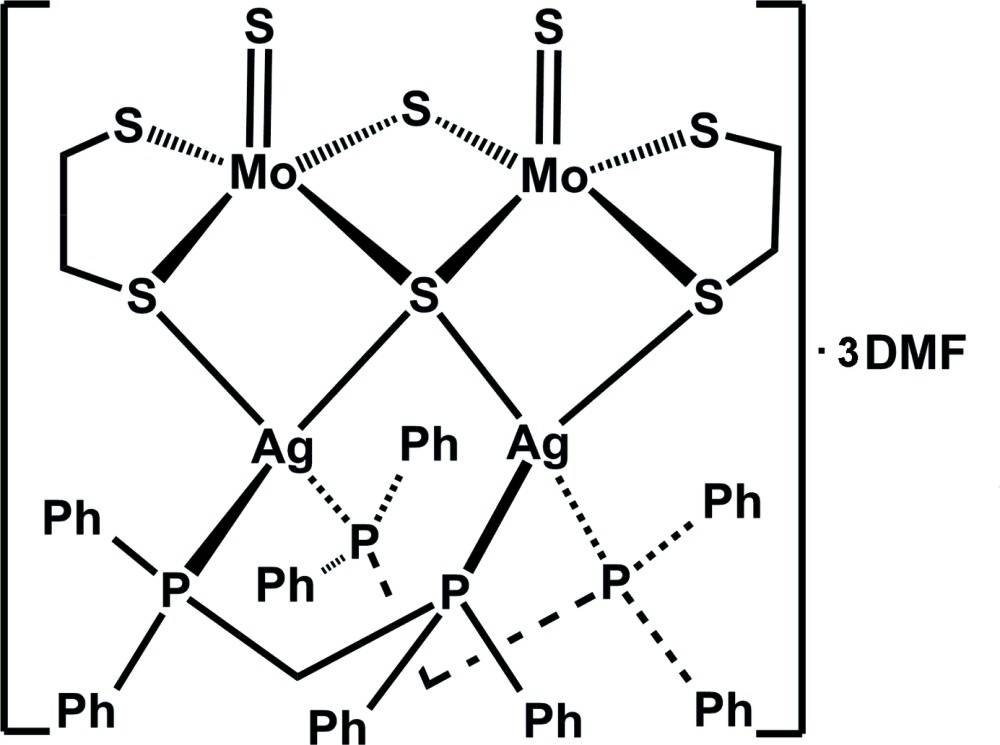


## Experimental

### 

#### Crystal data

[Ag_2_Mo_2_(C_2_H_4_S_2_)_2_S_4_(C_25_H_22_P_2_)_2_]·3C_3_H_7_NO
*M* *_r_* = 1708.3
Orthorhombic, 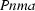

*a* = 26.022 (5) Å
*b* = 21.375 (4) Å
*c* = 12.721 (3) Å
*V* = 7076 (3) Å^3^
*Z* = 4
Mo *K*α radiation
μ = 1.26 mm^−1^
*T* = 223 K
0.35 × 0.20 × 0.07 mm

#### Data collection

Rigaku Mercury diffractometer
Absorption correction: multi-scan (*REQAB*; Jacobson, 1998 ▶) *T*_min_ = 0.746, *T*_max_ = 0.915
57121 measured reflections
6401 independent reflections
5931 reflections with *I* > 2σ(*I*)
*R*_int_ = 0.090

#### Refinement

*R*[*F* ^2^ > 2σ(*F* ^2^)] = 0.085
*wR*(*F* ^2^) = 0.188
*S* = 1.35
6401 reflections
401 parameters
H-atom parameters constrained
Δρ_max_ = 0.95 e Å^−3^
Δρ_min_ = −0.58 e Å^−3^

### 

Data collection: *CrystalClear* (Rigaku/MSC, 2001 ▶); cell refinement: *CrystalClear*; data reduction: *CrystalStructure* (Rigaku/MSC, 2004 ▶); program(s) used to solve structure: *SHELXS97* (Sheldrick, 2008 ▶); program(s) used to refine structure: *SHELXL97* (Sheldrick, 2008 ▶); molecular graphics: *SHELXTL* (Sheldrick, 2008 ▶); software used to prepare material for publication: *SHELXTL*.

## Supplementary Material

Crystal structure: contains datablocks I, global. DOI: 10.1107/S1600536809048648/ya2110sup1.cif

Structure factors: contains datablocks I. DOI: 10.1107/S1600536809048648/ya2110Isup2.hkl

Additional supplementary materials:  crystallographic information; 3D view; checkCIF report

## Figures and Tables

**Fig. 1.**
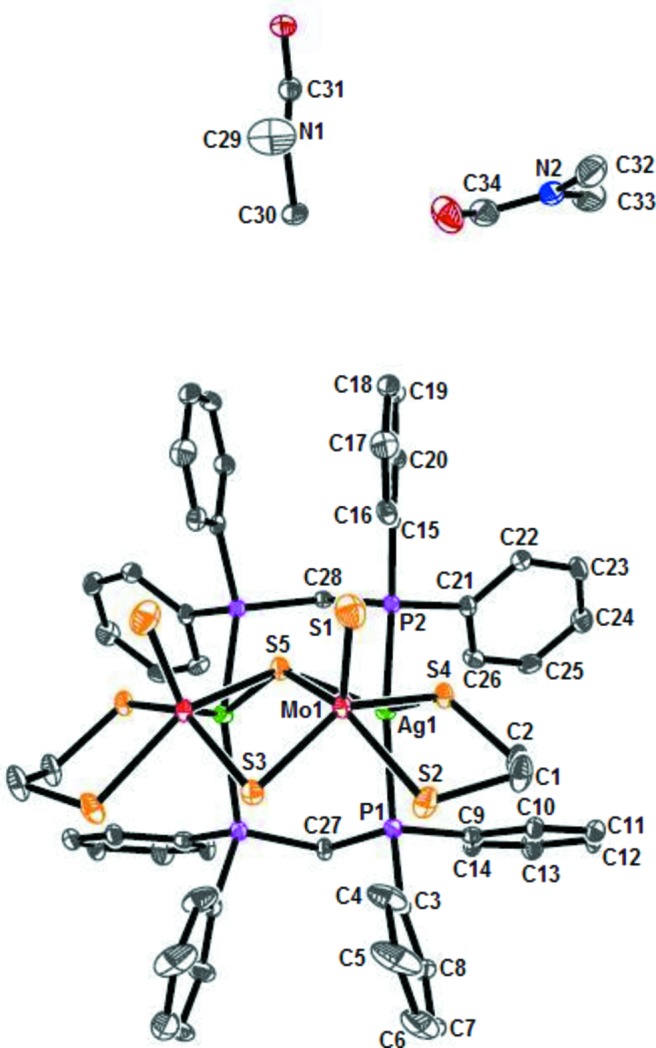
Molecular structure of 2.3DMF with thermal ellipsoids, drawn at 30% probability level; hydrogen atoms are omitted for clarity. The unlabeled atoms are derived from the reference atoms by means of the (x, 1/2 - y, z) symmetry transformation.

## supplementary crystallographic information

### Comment

In the past decades, the chemistry of the sulfido-bridged dinuclear clusters consisting of [*M*_2_S_4_] core (*M* = Mo, W) and various transition metals has attracted much attention. For example, precursors [(dtc)_2_Mo_2_S_2_(µ-S)_2_] (dtc = S_2_CNEt_2_) (Kuwata & Hidai, 2001) and [Cp*^x^*_2_Mo_2_S_2_(µ-S)_2_] (Cp*^x^* = pentamethyl-, pentaethyl- or pentabutyl-cyclopentadienyl) (Curtis *et al.*,1997; Halbert *et al.*, 1985; Kawaguchi *et al.*, 1997; Brunner *et al.*, 1985) were shown to react with transition metals to form both incomplete cubane-like [Mo_2_M'S_4_] and complete cubane-like [Mo_2_M'_2_S_4_] clusters. The type of cluster formed is dependent upon the ability of the terminal S_t_ and the bridging µ-S_b_ groups in [Mo_2_S_4_] core to bind further metal centers. Recently, another precursor [Et_4_N]_2_[(edt)_2_Mo_2_S_2_(µ-S)_2_] (1), which has a chelating edt at each Mo site of the [Mo_2_S_4_] core, has been introduced; its terminal S_t_, the doubly bridging µ-S_b_, and chelating S_edt_ are capable of binding Cu(I) centers (Zhu *et al.*,1990; Lin *et al.*, 1997; Wei *et al.*, 2008). However, until now, only quite limited data have been reported involving precursor 1 bound to Ag(I) complexes. In this paper we describe the result of our efforts to generate a Mo/Ag/S cluster [Mo_2_S_2_(µ-S)_2_(edt)_2_Ag_2_(dppm)_2_].3DMF (2.3DMF) by reaction of 1 with two equivalents of Ag(CH_3_CN)_4_ClO_4_ in the presence of dppm ligand.

The asymmetric unit of 2.3DMF contains half of the [Mo_2_S_2_(µ-S)_2_(edt)_2_Ag_2_(dppm)_2_] molecule, and one and a half DMF molecules (Fig. 1). The complex may be considered as having a [Mo_2_S_2_(µ-S)_2_(edt)_2_]^2-^ anionic unit bound to a [Ag_2_(dppm)_2_]^2+^ cation *via* two Ag-µ-S_edt_ and two Ag-µ_4_-S_b_ bonds. A crystallographic mirror plane runs through S3, S5, C27 and C28 atoms. Each Mo center has a distorted square pyramidal environment, consisting of one terminal S_t_, one S_edt_, one µ-S_edt_, and two µ-S atoms. Each Ag center has a distorted tetrahedral coordination made up of one µ-S_edt_, one µ_4_-S and two P atoms from two dppm ligands. The Ag1—S5 bond [2.924 (3) Å], involving the µ_4_-S atom, is much longer than Ag1—S4 with the S_edt_ atom [2.588 (2) Å]. The eight-membered [Ag—P—C—P—Ag—P—C—P] ring in the [Ag_2_(dppm)_2_]^2+ ^dication adopts a boat conformation. The Mo···Mo distance [2.8772 (14) Å] is longer than that in the precursor 1 [2.863 (3) Å] (Pan *et al.*,1984). The Mo1-µ-S4 bond length is elongated by 0.05 Å relative to that of Mo1—S2 as the S4 atom is involved in coordination to the Ag1 atom. The Mo1—S5 bond [2.344 (2) Å] is slightly longer than Mo1—S3 [2.322 (2) Å] due to the µ_4_-character of the S5 atom.

### Experimental

To a solution of 1 (76 mg, 0.1 mmol) in 10 ml of CH_2_Cl_2_ was added dropwise a solution of Ag(CH_3_CN)_4_ClO_4_ (29 mg, 0.2 mmol) in 20 ml of MeCN. A bulk of deep red precipitate was formed within s. The red slurry was stirred for 10 minutes, and a solution of dppm (79 mg, 0.2 mmol) in CH_2_Cl_2_ (10 ml) was added. The resulting mixture was stirred for 30 min, forming a homogenous red solution. Addition of MeOH to this solution yielded a red microcrystalline solid, which was collected by filtration, washed with MeCN and Et_2_O, and dried *in vacuo*. Recrystallization of the solid in DMF/*i*-PrOH afforded red crystals of 2.3DMF two days later. Yield: 63 mg (50% based on Mo).

### Refinement

Even though packing analysis shows solvent accessible voids, our attempts to locate additional solvent proved unsuccessful, and none of the geometrically placed atoms, centered around the void, could be reasonably refined. Because of the quick loss of crystallinity upon removal from the mother liquor, the structure has a limited accuracy with high *R*-factors and goodness of fit; optimization of weighting scheme results in high value of the second coefficient. Some of the phenyl atoms show intense thermal motion, however attempts to introduce disorder of the phenyl ring did not produce noticeable improvement of the accuracy of the model.

All H atoms were placed geometrically (C—H 0.93 Å for aromatic and formate, 0.96 Å and 0.97 Å for methyl and methylene groups respectively) and included in the refinement in the riding motion approximation with *U*_iso_(H) = 1.2*U*_eq_ of the parent atom [1.5*U*_eq_ for methyl groups].

### Crystal data

**Table:**

[Ag_2_Mo_2_(C_2_H_4_S_2_)_2_S_4_(C_25_H_22_P_2_)_2_]·3C_3_H_7_NO	*F*(000) = 3448
*M**_r_* = 1708.3	*D*_x_ = 1.604 Mg m^−^^3^
Orthorhombic, *P**n**m**a*	Mo *K*α radiation, λ = 0.71073 Å
Hall symbol: -P 2ac 2n	Cell parameters from 7734 reflections
*a* = 26.022 (5) Å	θ = 2.0–25.0°
*b* = 21.375 (4) Å	µ = 1.26 mm^−^^1^
*c* = 12.721 (3) Å	*T* = 223 K
*V* = 7076 (3) Å^3^	Platelet, red
*Z* = 4	0.35 × 0.20 × 0.07 mm

### Data collection

**Table:**

Rigaku Mercury diffractometer	6401 independent reflections
Radiation source: fine-focus sealed tube	5931 reflections with *I* > 2σ(*I*)
graphite	*R*_int_ = 0.090
ω scans	θ_max_ = 25.0°, θ_min_ = 3.4°
Absorption correction: multi-scan (REQAB; Jacobson, 1998)	*h* = −30→30
*T*_min_ = 0.746, *T*_max_ = 0.915	*k* = −25→25
57121 measured reflections	*l* = −15→15

### Refinement

**Table:**

Refinement on *F*^2^	Primary atom site location: structure-invariant direct methods
Least-squares matrix: full	Secondary atom site location: difference Fourier map
*R*[*F*^2^ > 2σ(*F*^2^)] = 0.085	Hydrogen site location: inferred from neighbouring sites
*wR*(*F*^2^) = 0.188	H-atom parameters constrained
*S* = 1.35	*w* = 1/[σ^2^(*F*_o_^2^) + (0.0747*P*)^2^ + 33.869*P*] where *P* = (*F*_o_^2^ + 2*F*_c_^2^)/3
6401 reflections	(Δ/σ)_max_ < 0.001
401 parameters	Δρ_max_ = 0.95 e Å^−^^3^
0 restraints	Δρ_min_ = −0.58 e Å^−^^3^

### Special details

**Table:**

Geometry. All e.s.d.'s (except the e.s.d. in the dihedral angle between two l.s. planes) are estimated using the full covariance matrix. The cell e.s.d.'s are taken into account individually in the estimation of e.s.d.'s in distances, angles and torsion angles; correlations between e.s.d.'s in cell parameters are only used when they are defined by crystal symmetry. An approximate (isotropic) treatment of cell e.s.d.'s is used for estimating e.s.d.'s involving l.s. planes.
Refinement. Refinement of *F*^2^ against ALL reflections. The weighted *R*-factor *wR* and goodness of fit *S* are based on *F*^2^, conventional *R*-factors *R* are based on *F*, with *F* set to zero for negative *F*^2^. The threshold expression of *F*^2^ > σ(*F*^2^) is used only for calculating *R*-factors(gt) *etc*. and is not relevant to the choice of reflections for refinement. *R*-factors based on *F*^2^ are statistically about twice as large as those based on *F*, and *R*- factors based on ALL data will be even larger.

### Fractional atomic coordinates and isotropic or equivalent isotropic displacement parameters (Å^2^)

**Table:**

	*x*	*y*	*z*	*U*_iso_*/*U*_eq_	Occ. (<1)
Ag1	0.00904 (2)	0.17618 (3)	0.71271 (5)	0.0236 (2)	
Mo1	0.12522 (3)	0.18270 (3)	0.92175 (5)	0.0211 (2)	
P1	−0.07622 (8)	0.17918 (10)	0.79382 (16)	0.0218 (5)	
S1	0.20195 (11)	0.16455 (14)	0.8910 (3)	0.0520 (7)	
O1	0.3659 (4)	0.2500	−0.0188 (7)	0.038 (2)	
N1	0.3070 (4)	0.2500	0.1140 (8)	0.025 (2)	
C1	0.0888 (5)	0.0364 (4)	0.9861 (8)	0.046 (3)	
H9A	0.1210	0.0199	0.9597	0.055*	
H9B	0.0743	0.0058	1.0341	0.055*	
C2	0.0528 (4)	0.0471 (4)	0.8971 (7)	0.038 (2)	
H16A	0.0192	0.0582	0.9243	0.045*	
H16B	0.0493	0.0089	0.8565	0.045*	
C3	−0.0801 (4)	0.1653 (4)	0.9351 (7)	0.028 (2)	
C4	−0.0361 (5)	0.1676 (6)	0.9945 (8)	0.056 (3)	
H8A	−0.0049	0.1781	0.9634	0.067*	
C5	−0.0384 (7)	0.1543 (8)	1.1014 (9)	0.086 (5)	
H1A	−0.0086	0.1561	1.1416	0.103*	
C6	−0.0835 (8)	0.1388 (6)	1.1481 (10)	0.083 (5)	
H2A	−0.0845	0.1296	1.2195	0.099*	
C7	−0.1276 (7)	0.1368 (6)	1.0894 (10)	0.071 (5)	
H6A	−0.1588	0.1270	1.1210	0.085*	
C8	−0.1255 (5)	0.1492 (5)	0.9838 (8)	0.044 (3)	
H5A	−0.1554	0.1468	0.9441	0.053*	
C9	−0.1156 (3)	0.1152 (4)	0.7415 (6)	0.0232 (18)	
C10	−0.0974 (4)	0.0548 (4)	0.7605 (7)	0.0274 (19)	
H22A	−0.0673	0.0489	0.7988	0.033*	
C11	−0.1241 (4)	0.0037 (4)	0.7223 (7)	0.034 (2)	
H29A	−0.1122	−0.0364	0.7365	0.041*	
C12	−0.1677 (4)	0.0114 (4)	0.6640 (8)	0.038 (2)	
H17A	−0.1854	−0.0231	0.6376	0.046*	
C13	−0.1850 (4)	0.0714 (5)	0.6449 (8)	0.040 (2)	
H13A	−0.2148	0.0771	0.6054	0.049*	
C14	−0.1595 (3)	0.1233 (4)	0.6829 (7)	0.031 (2)	
H20A	−0.1718	0.1633	0.6690	0.037*	
C15	0.0903 (3)	0.1697 (3)	0.4725 (6)	0.0202 (17)	
C16	0.1304 (3)	0.1645 (4)	0.5441 (7)	0.0269 (19)	
H26A	0.1235	0.1638	0.6158	0.032*	
C17	0.1805 (4)	0.1605 (5)	0.5084 (8)	0.040 (2)	
H7A	0.2071	0.1577	0.5569	0.048*	
C18	0.1916 (4)	0.1606 (4)	0.4042 (8)	0.035 (2)	
H14A	0.2255	0.1576	0.3817	0.042*	
C19	0.1521 (3)	0.1651 (4)	0.3318 (7)	0.031 (2)	
H30A	0.1596	0.1646	0.2603	0.037*	
C20	0.1019 (4)	0.1704 (4)	0.3646 (7)	0.0277 (19)	
H31A	0.0756	0.1745	0.3154	0.033*	
C21	−0.0123 (3)	0.1164 (4)	0.4566 (6)	0.0224 (17)	
C22	0.0119 (3)	0.0654 (4)	0.4086 (7)	0.029 (2)	
H25A	0.0475	0.0638	0.4033	0.035*	
C23	−0.0184 (4)	0.0170 (4)	0.3688 (7)	0.034 (2)	
H19A	−0.0027	−0.0173	0.3373	0.041*	
C24	−0.0713 (4)	0.0194 (4)	0.3758 (7)	0.036 (2)	
H18A	−0.0912	−0.0130	0.3488	0.043*	
C25	−0.0947 (4)	0.0702 (4)	0.4231 (7)	0.034 (2)	
H15A	−0.1304	0.0721	0.4270	0.041*	
C26	−0.0657 (3)	0.1177 (4)	0.4643 (7)	0.0282 (19)	
H27A	−0.0818	0.1511	0.4977	0.034*	
C27	−0.1134 (5)	0.2500	0.7688 (9)	0.024 (3)	
H33A	−0.1248	0.2500	0.6963	0.028*	
H33B	−0.1436	0.2500	0.8134	0.028*	
C28	0.0006 (4)	0.2500	0.4579 (8)	0.018 (2)	
H34A	0.0108	0.2500	0.3845	0.021*	
H34B	−0.0366	0.2500	0.4605	0.021*	
C29	0.3438 (7)	0.2500	0.1950 (14)	0.081 (7)	
H3A	0.3744	0.2296	0.1711	0.121*	0.50
H3B	0.3303	0.2281	0.2549	0.121*	0.50
H3C	0.3516	0.2923	0.2144	0.121*	0.50
C30	0.2532 (5)	0.2500	0.1436 (13)	0.042 (3)	
H30B	0.2436	0.2909	0.1677	0.063*	0.50
H30C	0.2477	0.2202	0.1989	0.063*	0.50
H30D	0.2326	0.2389	0.0839	0.063*	0.50
C31	0.3212 (5)	0.2500	0.0161 (11)	0.033 (3)	
H12	0.2950	0.2500	−0.0337	0.040*	
C32	0.2952 (5)	−0.0235 (6)	0.1897 (11)	0.067 (4)	
H4A	0.3174	−0.0415	0.1376	0.100*	
H4B	0.2776	−0.0563	0.2268	0.100*	
H4C	0.3152	0.0006	0.2384	0.100*	
C33	0.2240 (5)	−0.0106 (7)	0.0615 (11)	0.070 (4)	
H10A	0.2012	−0.0397	0.0950	0.105*	
H10B	0.2438	−0.0320	0.0090	0.105*	
H10C	0.2043	0.0220	0.0288	0.105*	
C34	0.2541 (4)	0.0767 (5)	0.1667 (10)	0.051 (3)	
H11	0.2296	0.1006	0.1316	0.061*	
P2	0.02494 (8)	0.17823 (9)	0.52142 (16)	0.0187 (4)	
S2	0.10053 (10)	0.11032 (11)	1.05640 (18)	0.0370 (6)	
N2	0.2582 (3)	0.0164 (4)	0.1394 (7)	0.042 (2)	
O2	0.2797 (3)	0.1029 (4)	0.2337 (7)	0.069 (3)	
S3	0.11821 (12)	0.2500	1.0644 (2)	0.0269 (7)	
S4	0.07638 (8)	0.10971 (9)	0.81247 (16)	0.0246 (5)	
S5	0.09599 (12)	0.2500	0.7891 (2)	0.0243 (6)	

### Atomic displacement parameters (Å^2^)

**Table:**

	*U*^11^	*U*^22^	*U*^33^	*U*^12^	*U*^13^	*U*^23^
Ag1	0.0258 (3)	0.0255 (3)	0.0194 (3)	0.0015 (3)	0.0011 (3)	0.0018 (3)
Mo1	0.0231 (4)	0.0186 (4)	0.0217 (4)	0.0017 (3)	−0.0047 (3)	0.0009 (3)
P1	0.0248 (11)	0.0197 (10)	0.0210 (11)	0.0013 (8)	0.0007 (9)	0.0021 (9)
S1	0.0415 (15)	0.0467 (16)	0.0678 (19)	0.0071 (12)	−0.0064 (14)	−0.0074 (14)
O1	0.038 (6)	0.040 (5)	0.035 (5)	0.000	0.010 (4)	0.000
N1	0.022 (5)	0.027 (5)	0.026 (6)	0.000	0.004 (4)	0.000
C1	0.074 (8)	0.027 (5)	0.036 (6)	−0.007 (5)	−0.017 (5)	0.010 (4)
C2	0.053 (6)	0.023 (5)	0.037 (5)	−0.002 (4)	−0.015 (5)	0.008 (4)
C3	0.040 (5)	0.020 (4)	0.024 (5)	0.008 (4)	0.003 (4)	0.002 (4)
C4	0.057 (7)	0.084 (9)	0.026 (5)	0.029 (6)	−0.001 (5)	−0.006 (6)
C5	0.111 (12)	0.130 (13)	0.018 (6)	0.066 (11)	−0.010 (7)	−0.005 (7)
C6	0.159 (16)	0.064 (9)	0.025 (6)	0.053 (10)	0.024 (9)	0.017 (6)
C7	0.131 (14)	0.043 (7)	0.039 (7)	−0.022 (8)	0.047 (8)	−0.006 (6)
C8	0.068 (7)	0.037 (6)	0.028 (5)	−0.015 (5)	0.018 (5)	−0.005 (4)
C9	0.025 (5)	0.029 (4)	0.015 (4)	0.000 (4)	0.002 (3)	0.000 (3)
C10	0.037 (5)	0.020 (4)	0.025 (5)	−0.002 (4)	0.002 (4)	0.000 (4)
C11	0.046 (6)	0.023 (4)	0.032 (5)	0.001 (4)	0.009 (5)	0.004 (4)
C12	0.055 (6)	0.018 (5)	0.042 (6)	−0.009 (4)	−0.002 (5)	−0.002 (4)
C13	0.035 (5)	0.035 (5)	0.052 (6)	−0.006 (4)	−0.008 (5)	−0.003 (5)
C14	0.034 (5)	0.021 (4)	0.037 (5)	0.002 (4)	−0.006 (4)	0.006 (4)
C15	0.022 (4)	0.014 (4)	0.025 (4)	0.000 (3)	−0.004 (3)	−0.002 (3)
C16	0.036 (5)	0.024 (4)	0.021 (4)	0.002 (4)	−0.006 (4)	−0.004 (3)
C17	0.033 (5)	0.044 (6)	0.044 (6)	0.003 (4)	−0.009 (5)	0.001 (5)
C18	0.025 (5)	0.029 (5)	0.050 (6)	0.000 (4)	0.009 (4)	0.000 (4)
C19	0.033 (5)	0.030 (5)	0.030 (5)	−0.005 (4)	0.007 (4)	0.002 (4)
C20	0.035 (5)	0.028 (5)	0.020 (4)	0.000 (4)	0.000 (4)	0.000 (4)
C21	0.029 (4)	0.019 (4)	0.019 (4)	0.001 (3)	−0.005 (4)	0.001 (3)
C22	0.028 (5)	0.027 (4)	0.032 (5)	−0.001 (4)	0.004 (4)	0.000 (4)
C23	0.046 (6)	0.023 (5)	0.032 (5)	0.000 (4)	0.000 (4)	−0.009 (4)
C24	0.040 (5)	0.034 (5)	0.034 (5)	−0.011 (4)	−0.006 (4)	−0.010 (4)
C25	0.028 (5)	0.042 (5)	0.033 (5)	−0.010 (4)	0.007 (4)	−0.003 (4)
C26	0.025 (5)	0.033 (5)	0.026 (5)	0.002 (4)	0.002 (4)	−0.007 (4)
C27	0.032 (7)	0.015 (5)	0.023 (6)	0.000	−0.004 (5)	0.000
C28	0.020 (6)	0.020 (5)	0.013 (5)	0.000	−0.001 (5)	0.000
C29	0.058 (12)	0.14 (2)	0.044 (10)	0.000	−0.026 (9)	0.000
C30	0.022 (7)	0.041 (8)	0.062 (10)	0.000	0.008 (7)	0.000
C31	0.029 (7)	0.032 (7)	0.039 (8)	0.000	−0.003 (6)	0.000
C32	0.048 (7)	0.057 (8)	0.095 (10)	−0.003 (6)	0.008 (7)	0.018 (7)
C33	0.048 (7)	0.080 (9)	0.082 (9)	−0.010 (7)	0.003 (7)	−0.023 (8)
C34	0.042 (6)	0.052 (7)	0.060 (7)	0.007 (5)	0.005 (6)	0.003 (6)
P2	0.0201 (10)	0.0189 (10)	0.0171 (10)	0.0009 (8)	0.0003 (8)	−0.0010 (8)
S2	0.0540 (15)	0.0328 (12)	0.0243 (12)	−0.0104 (11)	−0.0123 (11)	0.0080 (10)
N2	0.030 (4)	0.041 (5)	0.056 (6)	−0.007 (4)	0.008 (4)	−0.005 (4)
O2	0.061 (6)	0.064 (6)	0.080 (6)	0.000 (4)	−0.012 (5)	−0.032 (5)
S3	0.0349 (17)	0.0261 (15)	0.0197 (15)	0.000	−0.0050 (13)	0.000
S4	0.0325 (11)	0.0182 (10)	0.0229 (11)	0.0005 (8)	−0.0081 (9)	0.0003 (8)
S5	0.0351 (17)	0.0176 (14)	0.0203 (15)	0.000	−0.0031 (13)	0.000

### Geometric parameters (Å, °)

**Table:**

Ag1—P1	2.448 (2)	C16—C17	1.384 (13)
Ag1—P2	2.469 (2)	C16—H26A	0.9300
Ag1—S4	2.588 (2)	C17—C18	1.356 (14)
Ag1—S5	2.924 (3)	C17—H7A	0.9300
Ag1—Ag1^i^	3.1558 (14)	C18—C19	1.383 (13)
Mo1—S1	2.071 (3)	C18—H14A	0.9300
Mo1—S3	2.322 (2)	C19—C20	1.377 (13)
Mo1—S5	2.344 (2)	C19—H30A	0.9300
Mo1—S2	2.396 (2)	C20—H31A	0.9300
Mo1—S4	2.446 (2)	C21—C26	1.392 (12)
Mo1—Mo1^i^	2.8772 (14)	C21—C22	1.400 (12)
P1—C27	1.824 (7)	C21—P2	1.834 (8)
P1—C3	1.824 (9)	C22—C23	1.396 (12)
P1—C9	1.835 (8)	C22—H25A	0.9300
O1—C31	1.245 (15)	C23—C24	1.382 (13)
N1—C31	1.300 (16)	C23—H19A	0.9300
N1—C29	1.406 (18)	C24—C25	1.382 (13)
N1—C30	1.450 (16)	C24—H18A	0.9300
C1—C2	1.487 (13)	C25—C26	1.370 (12)
C1—S2	1.842 (10)	C25—H15A	0.9300
C1—H9A	0.9700	C26—H27A	0.9300
C1—H9B	0.9700	C27—P1^i^	1.824 (7)
C2—S4	1.824 (9)	C27—H33A	0.9700
C2—H16A	0.9700	C27—H33B	0.9700
C2—H16B	0.9700	C28—P2^i^	1.846 (6)
C3—C4	1.372 (15)	C28—P2	1.846 (6)
C3—C8	1.378 (13)	C28—H34A	0.9700
C4—C5	1.392 (16)	C28—H34B	0.9700
C4—H8A	0.9300	C29—H3A	0.9600
C5—C6	1.36 (2)	C29—H3B	0.9600
C5—H1A	0.9300	C29—H3C	0.9600
C6—C7	1.37 (2)	C30—H30B	0.9600
C6—H2A	0.9300	C30—H30C	0.9600
C7—C8	1.370 (16)	C30—H30D	0.9600
C7—H6A	0.9300	C31—H12	0.9300
C8—H5A	0.9300	C32—N2	1.437 (15)
C9—C14	1.373 (12)	C32—H4A	0.9600
C9—C10	1.396 (11)	C32—H4B	0.9600
C10—C11	1.382 (12)	C32—H4C	0.9600
C10—H22A	0.9300	C33—N2	1.452 (14)
C11—C12	1.365 (14)	C33—H10A	0.9600
C11—H29A	0.9300	C33—H10B	0.9600
C12—C13	1.382 (13)	C33—H10C	0.9600
C12—H17A	0.9300	C34—O2	1.218 (13)
C13—C14	1.381 (13)	C34—N2	1.339 (14)
C13—H13A	0.9300	C34—H11	0.9300
C14—H20A	0.9300	S3—Mo1^i^	2.322 (2)
C15—C16	1.389 (11)	S5—Mo1^i^	2.344 (2)
C15—C20	1.405 (12)	S5—Ag1^i^	2.924 (3)
C15—P2	1.820 (8)		
			
P1—Ag1—P2	124.54 (7)	C16—C17—H7A	119.3
P1—Ag1—S4	114.92 (7)	C17—C18—C19	119.5 (9)
P2—Ag1—S4	112.31 (7)	C17—C18—H14A	120.2
P1—Ag1—S5	123.16 (8)	C19—C18—H14A	120.2
P2—Ag1—S5	100.85 (8)	C20—C19—C18	120.6 (9)
S4—Ag1—S5	67.02 (6)	C20—C19—H30A	119.7
P1—Ag1—Ag1^i^	88.50 (5)	C18—C19—H30A	119.7
P2—Ag1—Ag1^i^	88.98 (5)	C19—C20—C15	119.9 (8)
S4—Ag1—Ag1^i^	123.29 (5)	C19—C20—H31A	120.1
S5—Ag1—Ag1^i^	57.35 (4)	C15—C20—H31A	120.1
S1—Mo1—S3	109.83 (12)	C26—C21—C22	119.7 (8)
S1—Mo1—S5	106.97 (12)	C26—C21—P2	118.8 (6)
S3—Mo1—S5	99.01 (8)	C22—C21—P2	121.2 (7)
S1—Mo1—S2	105.81 (11)	C23—C22—C21	118.8 (8)
S3—Mo1—S2	79.66 (8)	C23—C22—H25A	120.6
S5—Mo1—S2	145.47 (11)	C21—C22—H25A	120.6
S1—Mo1—S4	105.92 (10)	C24—C23—C22	120.8 (8)
S3—Mo1—S4	142.96 (10)	C24—C23—H19A	119.6
S5—Mo1—S4	79.26 (7)	C22—C23—H19A	119.6
S2—Mo1—S4	81.67 (8)	C23—C24—C25	119.7 (8)
S1—Mo1—Mo1^i^	100.80 (8)	C23—C24—H18A	120.1
S3—Mo1—Mo1^i^	51.72 (5)	C25—C24—H18A	120.1
S5—Mo1—Mo1^i^	52.15 (5)	C26—C25—C24	120.4 (9)
S2—Mo1—Mo1^i^	130.22 (6)	C26—C25—H15A	119.8
S4—Mo1—Mo1^i^	129.64 (5)	C24—C25—H15A	119.8
C27—P1—C3	106.1 (5)	C25—C26—C21	120.5 (8)
C27—P1—C9	105.0 (4)	C25—C26—H27A	119.7
C3—P1—C9	101.8 (4)	C21—C26—H27A	119.7
C27—P1—Ag1	115.4 (4)	P1^i^—C27—P1	112.2 (6)
C3—P1—Ag1	117.4 (3)	P1^i^—C27—H33A	109.2
C9—P1—Ag1	109.5 (3)	P1—C27—H33A	109.2
C31—N1—C29	120.6 (13)	P1^i^—C27—H33B	109.2
C31—N1—C30	121.6 (11)	P1—C27—H33B	109.2
C29—N1—C30	117.8 (13)	H33A—C27—H33B	107.9
C2—C1—S2	110.0 (7)	P2^i^—C28—P2	112.4 (6)
C2—C1—H9A	109.7	P2^i^—C28—H34A	109.1
S2—C1—H9A	109.7	P2—C28—H34A	109.1
C2—C1—H9B	109.7	P2^i^—C28—H34B	109.1
S2—C1—H9B	109.7	P2—C28—H34B	109.1
H9A—C1—H9B	108.2	H34A—C28—H34B	107.9
C1—C2—S4	110.5 (7)	N1—C29—H3A	109.5
C1—C2—H16A	109.5	N1—C29—H3B	109.5
S4—C2—H16A	109.5	H3A—C29—H3B	109.5
C1—C2—H16B	109.5	N1—C29—H3C	109.5
S4—C2—H16B	109.5	H3A—C29—H3C	109.5
H16A—C2—H16B	108.1	H3B—C29—H3C	109.5
C4—C3—C8	118.5 (9)	N1—C30—H30B	109.5
C4—C3—P1	119.4 (8)	N1—C30—H30C	109.5
C8—C3—P1	122.1 (8)	H30B—C30—H30C	109.5
C3—C4—C5	119.6 (13)	N1—C30—H30D	109.5
C3—C4—H8A	120.2	H30B—C30—H30D	109.5
C5—C4—H8A	120.2	H30C—C30—H30D	109.5
C6—C5—C4	121.0 (14)	O1—C31—N1	127.4 (13)
C6—C5—H1A	119.5	O1—C31—H12	116.3
C4—C5—H1A	119.5	N1—C31—H12	116.3
C5—C6—C7	119.6 (11)	N2—C32—H4A	109.5
C5—C6—H2A	120.2	N2—C32—H4B	109.5
C7—C6—H2A	120.2	H4A—C32—H4B	109.5
C8—C7—C6	119.7 (13)	N2—C32—H4C	109.5
C8—C7—H6A	120.2	H4A—C32—H4C	109.5
C6—C7—H6A	120.2	H4B—C32—H4C	109.5
C7—C8—C3	121.5 (12)	N2—C33—H10A	109.5
C7—C8—H5A	119.2	N2—C33—H10B	109.5
C3—C8—H5A	119.2	H10A—C33—H10B	109.5
C14—C9—C10	119.6 (8)	N2—C33—H10C	109.5
C14—C9—P1	124.5 (7)	H10A—C33—H10C	109.5
C10—C9—P1	115.9 (6)	H10B—C33—H10C	109.5
C11—C10—C9	119.9 (8)	O2—C34—N2	125.5 (11)
C11—C10—H22A	120.1	O2—C34—H11	117.3
C9—C10—H22A	120.1	N2—C34—H11	117.3
C12—C11—C10	121.0 (8)	C15—P2—C21	105.5 (4)
C12—C11—H29A	119.5	C15—P2—C28	104.7 (4)
C10—C11—H29A	119.5	C21—P2—C28	102.7 (4)
C11—C12—C13	118.5 (9)	C15—P2—Ag1	119.5 (3)
C11—C12—H17A	120.7	C21—P2—Ag1	110.0 (3)
C13—C12—H17A	120.7	C28—P2—Ag1	112.9 (3)
C14—C13—C12	121.8 (9)	C1—S2—Mo1	104.6 (3)
C14—C13—H13A	119.1	C34—N2—C32	120.7 (10)
C12—C13—H13A	119.1	C34—N2—C33	120.7 (10)
C9—C14—C13	119.3 (8)	C32—N2—C33	118.6 (10)
C9—C14—H20A	120.4	Mo1—S3—Mo1^i^	76.55 (10)
C13—C14—H20A	120.4	C2—S4—Mo1	107.9 (3)
C16—C15—C20	118.7 (8)	C2—S4—Ag1	117.7 (3)
C16—C15—P2	119.0 (6)	Mo1—S4—Ag1	106.28 (8)
C20—C15—P2	122.2 (6)	Mo1^i^—S5—Mo1	75.71 (9)
C17—C16—C15	119.8 (8)	Mo1^i^—S5—Ag1	145.22 (13)
C17—C16—H26A	120.1	Mo1—S5—Ag1	99.16 (6)
C15—C16—H26A	120.1	Mo1^i^—S5—Ag1^i^	99.16 (6)
C18—C17—C16	121.4 (9)	Mo1—S5—Ag1^i^	145.22 (13)
C18—C17—H7A	119.3	Ag1—S5—Ag1^i^	65.31 (7)

Symmetry codes: (i) *x*, −*y*+1/2, *z*.
